# The Effect of Short-Term Healthy Ketogenic Diet Ready-To-Eat Meals Versus Healthy Ketogenic Diet Counselling on Weight Loss in Overweight Adults: A Pilot Randomized Controlled Trial

**DOI:** 10.3390/nu17152541

**Published:** 2025-08-01

**Authors:** Melissa Hui Juan Tay, Qai Ven Yap, Su Lin Lim, Yuki Wei Yi Ong, Victoria Chantel Hui Ting Wee, Chin Meng Khoo

**Affiliations:** 1 Department of Dietetics, National University Hospital, Singapore 119074, Singapore; 2 Biostatistics Unit, Yong Loo Lin School of Medicine, National University Singapore, Singapore 117597, Singapore; qaiven@nus.edu.sg; 3 Asia Longevity Medical Centre, Singapore 238859, Singapore; limsulin01@gmail.com; 4 Nutrition and Dietetics, Health and Social Sciences, Singapore Institute of Technology, Singapore 828608, Singapore; 5 Division of Endocrinology, Department of Medicine, National University Hospital, Singapore 119074, Singapore; chin_meng_khoo@nuhs.edu.sg

**Keywords:** healthy ketogenic diet, weight loss, obesity, meal replacement, metabolic outcomes, Asian, adults

## Abstract

**Background/Objectives:** Conventional ketogenic diets, although effective for weight loss, often contain high total and saturated fat intake, which leads to increased low-density lipoprotein cholesterol (LDL-C). Thus, the Healthy Ketogenic Diet (HKD) was developed to address these concerns. It emphasizes calorie restriction, limiting net carbohydrate intake to 50 g per day, prioritizing unsaturated fats, and reducing saturated fat intake. However, adherence to the HKD remains a challenge in urban, time-constrained environments. Therefore, this pilot randomized controlled trial aimed to investigate the effects of Healthy Ketogenic Diet Ready-To-Eat (HKD-RTE) meals (provided for the first month only) versus HKD alone on weight loss and metabolic parameters among overweight adults. **Methods:** Multi-ethnic Asian adults (*n* = 50) with a body mass index (BMI) ≥ 27.5 kg/m^2^ were randomized into the HKD-RTE group (*n* = 24) and the HKD group (*n* = 26). Both groups followed the HKD for six months, with the HKD-RTE group receiving HKD-RTE meals during the first month. Five in-person workshops and mobile health coaching through the Nutritionist Buddy Keto app helped to facilitate dietary adherence. The primary outcome was the change in body weight at 6 months. Linear regression was performed on the change from baseline for each continuous outcome, adjusting for demographics and relevant covariates. Logistic regression was performed on binary weight loss ≥ 5%, adjusting for demographics and relevant covariates. **Results:** In the HKD group, participants’ adherence to the 50 g net carbohydrate target was 15 days, while that in the HKD-RTE group was 19 days over a period of 30 days. Participants’ adherence to calorie targets was 21 days in the HKD group and 23 days in the HKD-RTE. The average compliance with the HKD-RTE meals provided in the HKD-RTE group was 55%. The HKD-RTE group experienced a greater percentage weight loss at 1 month (−4.8 ± 3.0% vs. −1.8 ± 6.2%), although this was not statistically significant. This trend continued up to 6 months, with the HKD-RTE group showing a greater percentage weight reduction (−8.6 ± 6.8% vs. −3.9 ± 8.6%; *p* = 0.092). At 6 months, the HKD-RTE group had a greater reduction in total cholesterol (−0.54 ± 0.76 mmol/L vs. −0.05 ± 0.56 mmol/L; *p* = 0.283) and LDL-C (−0.43 ± 0.67 mmol/L vs. −0.03 ± 0.52 mmol/L; *p* = 0.374) compared to the HKD group. Additionally, the HKD-RTE group exhibited greater reductions in systolic blood pressure (−8.3 ± 9.7 mmHg vs. −5.3 ± 11.0 mmHg), diastolic blood pressure (−7.7 ± 8.8 mmHg vs. −2.0 ± 7.0 mmHg), and HbA1c (−0.3 ± 0.5% vs. −0.1 ± 0.4%) than the HKD group (not statistically significant for any). **Conclusions:** Both HKD-RTE and HKD led to weight loss and improved metabolic profiles. The HKD-RTE group tended to show more favorable outcomes. Short-term HKD-RTE meal provision may enhance initial weight loss, with sustained long-term effects.

## 1. Introduction

The Singapore National Population Health Survey 2022 highlighted an increase in the prevalence of obesity among adults aged 18–74, rising from 10.5% in 2020 to 11.6% in 2022 [1]. This trend is concerning, as obesity is a complex condition associated with various comorbidities, including diabetes mellitus and cardiovascular disease, imposing an economic burden on Singapore’s healthcare system [2,3,4,5,6,7].

Current weight loss guidelines recommend reducing dietary fat and overall caloric consumption to achieve a negative energy balance [8]. Various diets with differing macronutrient compositions have been introduced, such as the Atkins Diet, Weight Watchers Diet, South Beach Diet, Ornish Diet, and Zone Diet [9]. The ketogenic diet (KD) for weight loss is gaining popularity. It has a very low carbohydrate intake, moderate protein content, and high fat content, which induces nutritional ketosis and facilitates weight loss [10,11]. The diet promotes a metabolic shift toward fat oxidation and ketone body production, mimicking the physiological effects of prolonged fasting [12]. Additionally, its appetite-suppressing properties—attributable to ketone generation and stabilized blood glucose levels—may support sustained long-term adherence [13].

Studies have shown that the KD effectively induces short-term weight loss, particularly within 6 months to 1 year, among individuals with obesity [10,14,15,16]. The common drawbacks of KD are keto flu symptoms and nutrient deficiencies [17]. KD can also increase low-density lipoprotein cholesterol (LDL-C) levels due to its high content of saturated fats. High saturated fat and elevated LDL-C are risk factors for cardiovascular disease [18,19,20]. Therefore, the Healthy Ketogenic Diet (HKD) was designed to minimize the saturated fat content and the increase in circulating LDL-C [21]. It incorporates seven key components: (i) calorie restriction tailored to the individual, (ii) a 50 g net carbohydrate limit (total carbohydrate constituting 20–25% of calorie intake), (iii) low saturated and trans fats, (iv) total fat within 50% of calorie intake, emphasizing healthy fats such as monounsaturated fats and omega-3 fatty acids, (v) adequate protein (25–30% of calorie intake; 1.0–1.2 g/kg body weight), (vi) adequate fiber (20–30 g/day), and (vii) adequate fluids of at least 2 L per day [21].

A recent study has shown that HKD is more effective in promoting weight loss and improving metabolic profiles than an energy-restricted diet, without elevating LDL-C levels [21]. However, adherence to the KD or HKD is often challenging [22,23]. In Singapore and other Asian countries, carbohydrates account for more than 50% of daily energy intake, serving as the primary source of energy from food. The predominant eating-out culture and the widespread availability of high-calorie, high-fat, and sugar-rich foods may further contribute to rising obesity rates [2,24,25,26,27,28]. In addition, access to the HKD can be challenging outside the home environment. In the home environment, Buga et al. highlighted the burdensome need for self-prepared meals and careful food selection among busy individuals [29]; thus, a Ready-To-Eat (RTE) meal may offer greater convenience and support dietary adherence [30]. Previous studies on RTE have primarily focused on calorie-controlled meal replacements, but not the HKD [31,32,33,34,35].

In this pilot randomized controlled trial, we aimed to compare the effects of Healthy Ketogenic Diet Ready-To-Eat (HKD-RTE) meals versus following the HKD alone on weight loss and metabolic parameters in overweight individuals participating in a 6-month weight loss program.

## 2. Materials and Methods

### 2.1. Study Design

This study was a randomized controlled trial with an open-label, parallel-arm assignment. The study was approved by the National Healthcare Group Domain Specific Review Board in Singapore (DSRB Ref: 2023/00165) and prospectively registered on ClinicalTrials.gov (Identifier: NCT06022796). All participants provided written informed consent prior to participating in the study.

### 2.2. Study Participants

Participants were public healthcare employees recruited from the National University Hospital between March and August 2023 via an internal email broadcast containing a poster advertisement. The inclusion criteria were adults aged 21 to 75 years old, with a body mass index (BMI) of ≥27.5 kg/m^2^ [36], English literacy, and ownership of a smartphone. Exclusion criteria included pre-existing heart disease, untreated endocrinopathies (e.g., hyperthyroidism or hypothyroidism), advanced kidney disease, severe cognitive or psychiatric disorders, active malignancy, use of medications that affect appetite or weight, prior or planned bariatric surgery, current pregnancy or intent to become pregnant, or current lactation. Participants with diabetes who were on insulin therapy were also excluded due to the risk of hypoglycemia.

### 2.3. Run-In Period

Eligible participants who completed the two-week run-in period were instructed to log their dietary intake and physical activity consistently and to measure their weight twice a week using the Nutritionist Buddy (nBuddy) Keto app (Heartvoice Pte Ltd., Singapore) [37,38,39,40]. The purpose of the run-in phase was to identify and exclude individuals who were non-adherent or not proficient with the app, thereby minimizing data loss and enhancing the integrity of subsequent data analysis.

### 2.4. Randomization and Masking

Eligible participants were randomized into either the control (HKD) or intervention (HKD-RTE) group in a 1:1 allocation ratio. RStudio version 3.6.3 was used to generate randomization codes with blocks of four, stratified by gender and BMI category (<35 and ≥35 kg/m^2^). Stratification by BMI was important, as individuals with a BMI ≥ 35 kg/m^2^ are classified as having Class III obesity, which is associated with a higher risk of chronic health conditions. This stratification ensured a more balanced distribution of participants into the control and intervention groups, thereby reducing potential confounding related to obesity-associated variables. Allocation was performed by a third party not involved in the study, using sequentially numbered, opaque, sealed envelopes. The envelopes were opened in sequence by the investigator after participant consent, and the group assignment was revealed accordingly. Blinding of participants and investigators was not feasible due to the nature of the intervention.

### 2.5. Intervention

**Figure 1:**
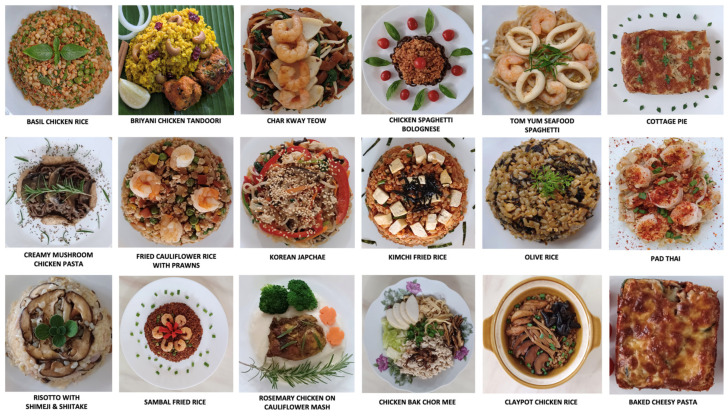
The 18 varieties of HKD-RTE meals (adapted from [41]).

The diet intervention was conducted over six months. Both the HKD and HKD-RTE groups were instructed to follow the HKD Guidelines, which prescribed an energy-restricted diet low in saturated and trans fats, with a daily net carbohydrate intake capped at 50 g [21]. In the HKD-RTE group, the participants were provided with one month of portion-controlled RTE meals (HealthFull; HealthFull Pte Ltd., Singapore) for both lunch and dinner [41] (Figure 1). The HKD-RTE meals were self-selected weekly at the clinic from a menu of 18 options and were provided at no cost. Each HKD-RTE meal provided an average of 316 kcal, 15 g of net carbs, 25 g of protein, and 7 g of fiber. HKD-RTE participants were also advised to choose low-carbohydrate breakfast and snack options to remain within the 50 g net carbohydrate limit per day.

In the HKD group, participants were taught to self-select or prepare their meals using the relevant food groups discussed during the workshops.

All participants attended a total of five workshops, conducted in person, over the six-month intervention period. The first and second workshops were conducted separately for each group to support a smoother transition for the HKD-RTE group from one month of RTE meals to a self-administered HKD. Subsequent workshops were conducted jointly for all participants. Each session, led by a dietitian, lasted approximately one hour and addressed key nutrition topics, including the following: (i) the fundamentals of the HKD and the use of technology and physical activity; (ii) strategies for food selection at home and when dining out; (iii) a supermarket tour and guidance on food labeling; (iv) overcoming weight plateaus; and (v) behavior change strategies and tips for maintaining weight and following dietary recommendations after the intervention.

All participants used the nBuddy Keto app [21,37,38,39,40] to track their weight twice a week and log their daily dietary intake and physical activity. Individualized calorie limits were automatically calculated based on body weight, gender, age, and activity levels. Participants were encouraged to log their meals via the app to stay within their assigned calorie and 50 g net carbohydrate limits. Participants were encouraged to increase their step counts from 3000 (first week) to 7000 (second week) and then to 10,000 (third week onwards), as tolerated. Weekly educational videos were provided through the app throughout the six month intervention, covering topics such as weight management, healthy keto meal planning, behavioral strategies, and physical activity. Multivitamin mineral supplementation was also introduced to all participants in the second month. This prevents vitamin and mineral deficiencies, maintains a healthy electrolyte homeostasis, and supports immune function and antioxidant defense mechanism [11]. Participants were also encouraged to consume at least 2 L of unsweetened fluids daily to prevent dehydration.

Health coaching was tailored to each participant’s input on the app. This process was made accessible to the research dietitian via the app’s dashboard. Through individualized virtual interactions via the app’s chat function, the research dietitian facilitated behavioral change by regularly reviewing the participants’ food intake, step count, and weight. Real-time feedback and motivational interviewing skills were utilized to help participants overcome barriers to change [40,42].

### 2.6. Outcome Evaluation

The primary outcome was mean weight change from baseline at 6 months of diet intervention. Secondary outcomes included mean weight change from baseline at 1 month and 3 months, as well as changes in metabolic profiles, including total cholesterol (TC), triglycerides (TG), and LDL-C, HDL-cholesterol (HDL-C), glycated hemoglobin (HbA1c), Fasting Blood Glucose (FBG), alanine transaminase (ALT), aspartate transaminase (AST), systolic blood pressure (SBP), and diastolic blood pressure (DBP) at both 3 and 6 months. Nutrient intake derived from the nBuddy Keto app was assessed at 1, 3, and 6 months. Adherence to the 50 g net carbohydrate and calorie limits was evaluated based on dietary logs submitted through the app. Compliance with the HKD-RTE meals was defined as the logging of both lunch and dinner meals in the nBuddy Keto App throughout the first month. It was assumed that the participants consumed meals logged in the app.

### 2.7. Anthropometric and Biochemical Measurements

During study visits, participants’ body weight was measured using a digital weighing scale (Omron HN-289, Omron Healthcare Co., Ltd., Kyoto, Japan), which had been calibrated using standardized weights. Measurements were taken with participants wearing light clothing and without shoes. Blood pressure was measured using an automated monitor (Omron HBP-1300, Omron Healthcare Co., Ltd., Kyoto, Japan). For both body weight and blood pressure, the average of two measurements was used. Fasting blood samples were collected following an overnight fast and were analyzed at the National University Hospital Referral Laboratory.

Participants attended follow-up visits at 1, 3, and 6 months post-enrollment for repeated body weight measurements, with blood tests conducted at 3 and 6 months. At each time point, two-day food diaries were obtained from the nBuddy Keto app to assess nutrient intake. A dietitian analyzed dietary intake using the localized nutrient analysis platform integrated into the nBuddy Keto app. This platform draws on several food composition databases, including the Singapore Energy and Nutrient Composition of Food, the Malaysian Food Composition database, and the United States Department of Agriculture (USDA) database, as well as nutritional information from food packaging and recipes.

### 2.8. Sample Size

The sample size was calculated based on the assumption of at least a large Cohen’s effect size of 0.9 for the difference in weight loss at 6 months between groups. A minimum sample size of 20 participants per group would provide 80% power at a 0.05 level of significance (two-sided). A total sample size of 50 participants (25 per arm) was planned, factoring in a 10% attrition rate.

### 2.9. Statistical Analysis

All statistical analyses were performed using IBM SPSS Statistics (version 29, IBM Corporation, Armonk, NY, USA). Differences in continuous variables were assessed using a 2-sample *t*-test, while Chi-square or Fisher’s exact test was used for categorical variables. Linear regression was performed on the change from baseline for each continuous outcome, adjusting for demographics and relevant covariates. Type 1 errors for multiple comparisons were adjusted using the Benjamini–Hochberg procedure, with a false discovery rate of 0.20. A comparison of changes from baseline was performed using a paired Student *t*-test. Logistic regression was performed to analyze the binary outcome of weight loss of ≥5%, adjusting for demographics and relevant covariates. Statistical significance was set at *p* < 0.05.

## 3. Results

### 3.1. Study Participants

**Figure 2:**
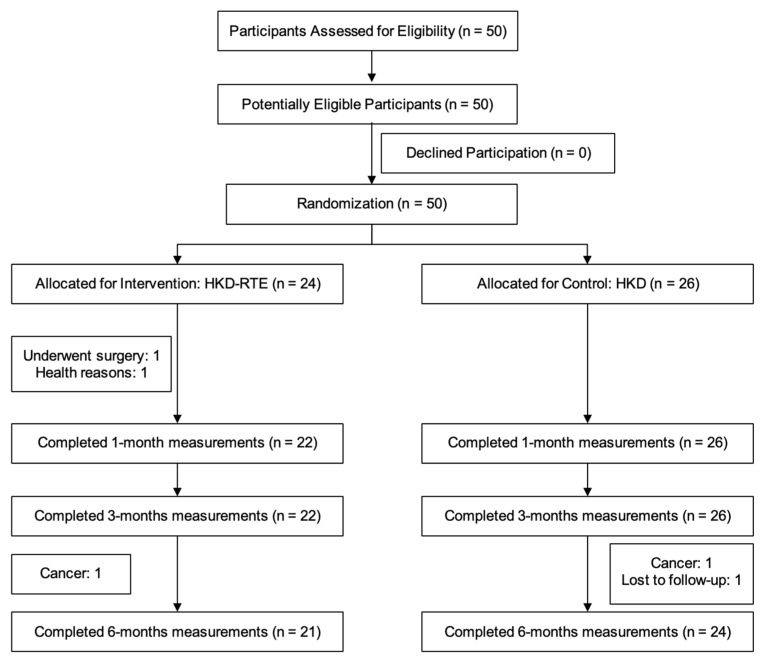
Participant flowchart.

A total of 50 participants were screened, with all enrolled and randomized to either the HKD-RTE (*n* = 24) or the HKD group (*n* = 26) (Figure 2).

### 3.2. Baseline Characteristics

Table 1 summarizes the participants’ baseline characteristics. Overall, baseline characteristics were similar between the HKD-RTE and HKD groups.

### 3.3. Adherence to Diet Prescription

In the HKD group, participants’ adherence to the 50 g net carbohydrate target was 15 days, while that in the HKD-RTE group was 19 days over a period of 30 days. Participants’ adherence to calorie targets was 21 days in the HKD group and 23 days in the HKD-RTE. The average compliance with the HKD-RTE meals provided in the HKD-RTE group was 55%.

### 3.4. Changes in the Body Weight and Metabolic Parameters

**Figure 3:**
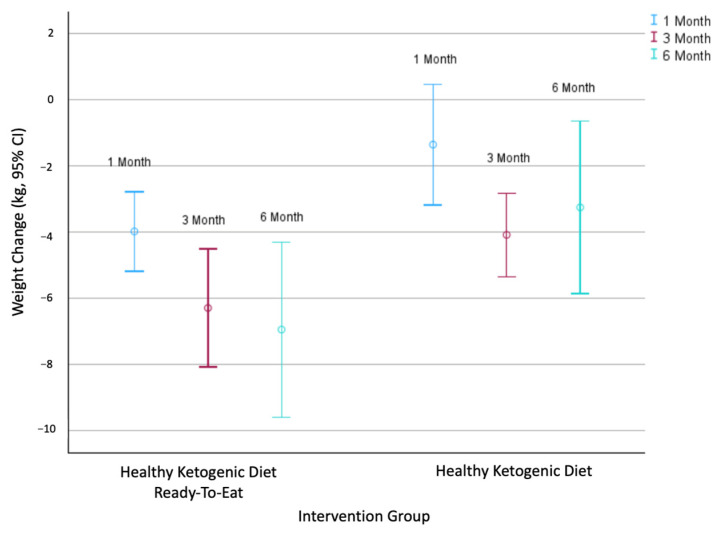
The 95% confidence intervals for body weight (kg) change at 1, 3, and 6 months in the HKD-RTE group and HKD group.

Table 2 shows the changes in weight and metabolic parameters between groups. At 1, 3, and 6 months, the HKD-RTE group experienced greater reductions in body weight compared to the HKD group (Figure 3); however, these mean differences were not statistically significant (Table 2). At 3 months, both the HKD-RTE and HKD groups achieved clinically significant weight loss of ≥5%, with 7.6 ± 4.6% and 5.3 ± 3.6%, respectively (Table 2). The HKD-RTE group maintained this clinically significant weight loss at 6 months (8.6 ± 6.8%), whereas the HKD group did not (3.9 ± 8.6%). At 1 month, the HKD-RTE group had 4.3 times (95% CI 0.9–20.2, *p* = 0.069) greater odds of achieving ≥5% weight loss compared to the HKD group; however, this difference was not statistically significant (Table 3).

**Figure 4:**
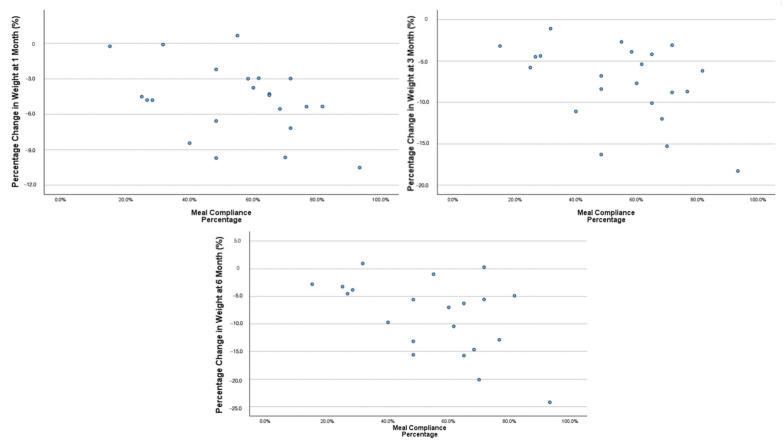
Association between the first month of HKD-RTE meal compliance and percent change in body weight (%) at 1, 3, and 6 months.

Figure 4 revealed that the majority of HKD-RTE participants complied with the HKD-RTE meals 60–80% of the time during the first month. At 1, 3, and 6 months, participants with higher compliance demonstrated greater and more consistent weight loss. In contrast, those with lower compliance exhibited more variable patterns and inconsistent changes in weight, including minimal weight loss or even weight gain. Each 1% increase in compliance was associated with an additional 0.109 kg of weight loss over 3 months (95% CI: −0.218–0.001; *p* = 0.049) (Table 4). At 1 month, HKD-RTE participants with a meal compliance rate of 60% or higher achieved weight loss ranging from approximately 3% to 10% (Figure 4).

### 3.5. Cardiometabolic Outcomes

Table 2 shows the changes in the cardiometabolic outcomes between the two intervention groups. At 3 months, the HKD-RTE group exhibited greater reductions in systolic blood pressure (−10.2 ± 12.7 mmHg vs. −6.7 ± 10.5 mmHg), diastolic blood pressure (−8.3 ± 6.8 mmHg vs. −5.3 ± 8.2 mmHg), HbA1c (−0.4 ± 0.6% vs. −0.3 ± 0.3%), and Fasting Blood Glucose (FBG) (−0.4 ± 0.7 mmol/L vs. −0.2 ± 0.5 mmol/L) than the HKD group, but this was not statistically significant. This trend persisted for 6 months.

At 6 months, the HKD-RTE group also exhibited greater reductions in TC (−0.54 ± 0.76 mmol/L vs. −0.05 ± 0.56 mmol/L), TG (−0.26 ± 0.44 mmol/L vs. −0.13 ± 0.35 mmol/L), and LDL-C (−0.43 ± 0.67 mmol/L vs. −0.03 ± 0.52 mmol/L) compared to the HKD group (not statistically significant for any). Moreover, HKD-RTE achieved statistically significant within-group reductions in TC and LDL-C at both 3 months and 6 months (*p* < 0.05).

### 3.6. Dietary Intake

Overall, both the HKD and HKD-RTE groups demonstrated reductions in nutrient intake (Table 2). At 1 month, the HKD group showed greater reductions across all nutrients compared to the HKD-RTE group, a trend that remained generally consistent over time. At 1 month, all nutrients except saturated fat, dietary cholesterol, trans fat, and net carbohydrates exhibited statistically significant between-group differences (*p* < 0.05). At 1 month, the HKD-RTE group had significantly higher intakes of protein (1.8 ± 15.1 g), polyunsaturated fatty acids (PUFA) (0.4 ± 4.0 g), and fiber (5.0 ± 6.2 g) compared to the HKD group. At 3 months, the reduction in dietary cholesterol was greater in the HKD-RTE group compared to the HKD group (−119.63 ± 190.38 mg vs. −30.85 ± 269.06 mg, *p* = 0.078), although this difference was not statistically significant. This trend persisted for 6 months.

## 4. Discussion

In this randomized controlled study, we showed that HKD-RTE induced greater weight loss than HKD alone, despite providing RTE only for the initial month of the 6 month intervention. HKD-RTE resulted in 4.3 times higher odds of achieving 5% weight loss in the first month of diet intervention. We also confirmed that weight loss with HKD, with or without RTE, improves metabolic profiles, including blood pressure, blood sugar, and lipid profiles, with a greater improvement in the HKD-RTE group.

Participants in the HKD-RTE group achieved a mean weight loss of 3.9 kg (4.8%) within the first month, approaching the clinically significant threshold of 5% weight loss [43]. These findings align with a previous study reporting an approximate 2 kg weight loss over two weeks with two short-term meal replacements [34]. The clear visual representation of portion sizes and nutrient balance in HKD-RTE meals might have helped participants transition more easily to self-prepared meals [33,44,45]. Additionally, the weight loss in the first month may have enhanced participants’ motivation and adherence through improved physical appearance, body image, mobility, energy levels, and perceived health [27,34,46,47,48,49,50]. These effects likely contributed to the sustained mean weight reduction of −7.0 ± 5.8 kg at 6 months. Comparatively, the participants in the HKD group had less weight loss than the HKD-RTE group. Several factors could have contributed to the lack of statistical significance between groups at all time points, including the small sample size, the established efficacy of the HKD [21], the provision of five workshops and health coaching via the nBuddy Keto app to both groups, and the limited duration of HKD-RTE meal provision, which lasted only one month. Interestingly, we observed a weight regain from 3 months (−4.4 ± 3.1 kg) to 6 months (−3.3 ± 6.2 kg) in the HKD group; however, the weight loss was sustained in the HKD-RTE group. Metabolic adaptations and declining adherence to diet over time may explain the weight regain [17].

In the first month, the average compliance rate with HKD-RTE meals was 55%. Participants in the HKD-RTE group demonstrated greater adherence to the 50 g net carbohydrate limit (19 days vs. 15 days) and calorie restriction (23 days vs. 21 days) compared to the HKD group. These findings suggest even small differences in compliance with CHO and calorie restriction with HKD-RTE meals can enhance overall dietary adherence and contribute to greater and sustained weight loss over six months. In addition, the greater dietary variability in the HKD group may have led to an overestimation of adherence. Although a recent systematic review reported dietary adherence rates exceeding 90% with medically tailored meals [51], the present study observed HKD-RTE meal compliance to be 60–80%. This level of compliance is considered practical in real-world settings, where routine meal consumption is often disrupted by minor illnesses, travel, or social events [35]. Several factors, such as dietary monotony, emotional eating, individual food preferences, and discrepancies between participants’ intentions and actual eating behavior, may have further influenced HKD-RTE meal compliance [52,53]. Shared mealtimes with others may also affect one’s decision to consume RTE meals [54]. Additionally, some participants who traveled might have postponed the RTE meals and consumed them beyond the initial one month period. However, this study did not conduct systematic tracking, which future research should consider implementing. Consistent with previous studies [55], our findings showed that a higher compliance with HKD-RTE meals was associated with greater weight loss. Each 1% increase in compliance was associated with an additional 0.109 kg of weight loss over 3 months. Future studies should investigate the factors that increase compliance in dietary interventions to optimize weight loss outcomes.

Despite achieving greater weight loss, the HKD-RTE group had smaller reductions in nutrient intake across most time points compared to the HKD group. Kuriyan et al. [34] reported a similar trend, finding that the intervention group that consumed a low-fat cereal meal replacement twice daily lost more weight, despite both groups showing comparable reductions in energy intake. Individuals attempting weight loss often underreport their intake, which may explain this discrepancy [56,57]. Additionally, the greater dietary variability within the HKD group may have contributed to the under-reporting. In contrast, the standardized composition of the HKD-RTE meals likely facilitated more consistent and accurate dietary reporting within the HKD-RTE group.

During the first month, the HKD-RTE group demonstrated increased intake of desirable nutrients, including protein, PUFA, and fiber. Protein and fiber enhance satiety and reduce overall energy intake [58,59]. PUFA intake increases fat oxidation and reduces fat mass [60]. Although our study did not observe an increase in monounsaturated fatty acid (MUFA) intake in the HKD-RTE group at 1 month, the reduction (−0.7 ± 5.8 g) was minimal compared to the HKD group (−8.0 ± 9.0 g). Participants’ efforts to keep within their calorie limit for weight loss may have contributed to the slight reduction. The higher MUFA energy proportion in the HKD-RTE group is desirable. MUFA supports improved glucose metabolism and insulin sensitivity, which contribute to weight loss [61]. Our findings highlight the potential of appropriately formulated HKD-RTE meals to enhance weight loss.

Both HKD and HKD-RTE demonstrated improvements in blood pressure, blood sugar, and lipid profiles. The HKD-RTE group showed greater improvements, along with greater weight loss, compared to the HKD group. Previous studies have established this association [62,63]. Moreover, Rock et al. [35] reported that consuming energy-restricted, pre-packaged meals twice a day for three months resulted in greater weight loss (~8%), improved lipid profiles, and lower blood pressure compared to a self-selected diet (weight loss of 6%). Similarly, in our study, the HKD-RTE group achieved a 7.6% weight loss and showed improvements in blood pressure and lipid profiles at 3 months. This outcome suggests that consuming HKD-RTE meals twice daily for a month can lead to effective weight loss and improved metabolic profiles.

Considering that approximately 60% of Singaporeans dine out at least four times a week [64], the limited availability of nutritious options, time constraints, low motivation, and inconvenience of meal preparation, HKD-RTE meals can serve as a structured dietary intervention [29,65]. This study is the first to evaluate the impact of HKD-RTE meals on weight loss with technology-based health coaching. The stratified randomized controlled trial design enhances internal validity by minimizing selection bias and ensuring a balanced distribution of known confounders, thereby increasing the reliability of the findings. Additionally, the integration of in-person workshops with mobile health technology (via the nBuddy Keto app) facilitated real-time monitoring and personalized coaching, reinforcing behavior change, improving diet adherence, and boosting participants’ self-efficacy and confidence [66]. These findings are particularly relevant to urban Asian populations, where time constraints and frequent dining out present ongoing challenges to maintaining consistent dietary habits.

The present study has several limitations. The six months intervention included the HKD-RTE meals only during the first month. Participants were also not blinded to group assignments, which may have influenced compliance and increased the likelihood of dietary crossover. The small sample size and single-center design limit the generalizability of the findings. Reliance on self-reported dietary intake via the nBuddy Keto app introduces potential inaccuracies in reporting. Moreover, the sample comprised public healthcare employees, who may not be representative of the general population, further restricting the external validity of the findings. Future research should consider evaluating the effects of HKD-RTE meals in controlled clinical settings and with more diverse populations. A comprehensive meal replacement protocol—including breakfast and snacks, a structured weight maintenance phase, and objective measures such as ketone levels, body composition measurements (e.g., waist, hip, and thigh circumferences), and gut microbiome profiling—is recommended to provide more in-depth insights. Additionally, strategies to promote sustainable use of HKD-RTE meals may help enhance long-term compliance.

## 5. Conclusions

In conclusion, both HKD-RTE and HKD led to weight loss and improved metabolic profiles. The HKD-RTE group tended to show more favorable outcomes. HKD-RTE may serve as an effective strategy in settings where limited time and cooking skills hinder dietary adherence. A larger-scale study will be needed to confirm the findings, assess participants’ satisfaction, and evaluate the long-term acceptability of HKD-RTE meals.

## Figures and Tables

**Table 1:** Baseline characteristics of study participants.

Variable	HKD-RTE (*n* = 24)	HKD (*n* = 26)	*p* Value ^a^
Gender, *n* (%)			
Female	20 (83.3%)	22 (84.6%)	1.000
Male	4 (16.7%)	4 (15.4%)	
Ethnicity, *n* (%)			
Chinese	13 (54.2%)	18 (69.2%)	0.633
Malay	2 (8.3%)	1 (3.8%)	
Indian	5 (20.8%)	3 (11.5%)	
Others	4 (16.7%)	4 (15.4%)	
Age (years)			
Mean	43.3 ± 7.3	39.0 ± 10.7	0.111
Range	26–54	24–69	
Weight, kg/m^2^	81.7 ± 15.8	83.4 ± 13.2	0.685
Body Mass Index, kg/m^2^	31.6 ± 4.6	32.2 ± 4.4	0.691
Systolic blood pressure, mmHg	126.6 ± 12.9	122.4 ± 14.3	0.272
Diastolic blood pressure, mmHg	83.1 ± 8.3	77.7 ± 9.9	0.042
HbA1c, %	6.0 ± 1.1	5.7 ± 0.6	0.391
Fasting blood glucose, mmol/L	5.7 ± 1.6	5.4 ± 0.9	0.413
Total cholesterol, mmol/L	5.2 ± 1.1	5.0 ± 0.7	0.487
Triglyceride, mmol/L	1.4 ± 0.8	1.2 ± 0.4	0.425
LDL cholesterol, mmol/L	3.3 ± 1.0	3.3 ± 0.7	0.918
HDL cholesterol, mmol/L	1.3 ± 0.2	1.2 ± 0.2	0.310
Co-morbidity, *n* (%)			
Hypertension			
No	8 (33.3%)	14 (53.8%)	0.144
Yes	16 (66.7%)	12 (46.2%)	
Diabetes			
No	18 (75.0%)	21 (80.8%)	0.623
Yes	6 (25.0%)	5 (19.2%)	
Hyperlipidemia			
No	4 (16.7%)	4 (15.4%)	1.000
Yes	20 (83.3%)	22 (84.6%)	
Nutrient intake			
Calorie, kcal	1229 ± 372	1422 ± 464	0.114
Protein, g	64.1 ± 20.8	69.8 ± 27.1	0.416
Total fat, g	52.5 ± 18.0	60.6 ± 26.5	0.219
Saturated fat, g	19.9 ± 8.9	23.2 ± 12.0	0.275
MUFA, g	18.9 ± 5.9	21.9 ± 11.4	0.237
PUFA, g	9.3 ± 3.5	10.3 ± 4.2	0.392
Trans fat, g	0.2 ± 0.2	0.2 ± 0.3	0.379
Carbohydrate, g	126.0 ± 54.3	150.1 ± 38.7	0.075
Net Carbohydrate, g	114.8 ± 53.1	138.2 ± 37.4	0.077
Sugar, g	31.4 ± 21.1	35.2 ± 14.4	0.452
Fiber, g	11.1 ± 4.5	11.9 ± 3.7	0.463
Sodium, mg	2358 ± 962	2514 ± 1193	0.614
Step count	7796.3 ± 3544.5	6409.4 ± 3915.7	0.204

Data expressed as mean ± SD for continuous variables; absolute numbers (percentage) for categorical variables. HbA1c, Glycated hemoglobin; LDL, Low-density Lipoprotein; HDL, High-density Lipoprotein; MUFA, Monounsaturated fatty acid; PUFA, Polyunsaturated fatty acid. ^a^ Chi-square, Fisher exact, independent samples *t*-test as appropriate.

**Table 2:** Primary and secondary outcomes at 1, 3, and 6 months after enrollment.

				Unadjusted			Adjusted		
Outcomes	*n*	Mean Change from Baseline		Between-Group Differences			Between-Group Differences ^a^		
		HKD-RTE (*n* = 24)	HKD (*n* = 26)	Mean Difference (95% CI)	*p* Value	Cohen d	Mean Difference (95% CI)	*p* Value	Cohen d
∆ Weight, kg									
1 month	48	−3.9 ± 2.6 *	−1.7 ± 4.3	−2.2 (−4.3–−0.1)	0.040	0.63	−1.8 (−3.8–0.2)	0.078	0.26
3 month	48	−6.1 ± 3.9 *	−4.4 ± 3.1 *	−1.7 (−3.8–0.3)	0.096	0.49	−1.5 (−4.0–1.0)	0.245	0.18
6 month	45	−7.0 ± 5.8 *	−3.3 ± 6.2 *	−3.7 (−7.3–−0.1)	0.046	0.62	−4.7 (−10.3–0.9)	0.096	0.26
∆ Weight, %									
1 month	48	−4.8 ± 3.0	−1.8 ± 6.2	−3.0 (−6.0–−0.2)	0.040	0.63	−2.4 (−5.4–0.5) ^b^	0.106 ^b^	0.24 ^b^
3 month	48	−7.6 ± 4.6	−5.3 ± 3.6	−2.3 (−4.7–0.1)	0.057	0.56	−1.7 (−4.5–1.1) ^b^	0.223 ^b^	0.18 ^b^
6 month	45	−8.6 ± 6.8	−3.9 ± 8.6	−4.7 (−9.4–−0.0)	0.049	0.61	−6.2 (−13.4–1.1) ^b^	0.092 ^b^	0.27 ^b^
∆ BMI, kg/m^2^									
1 month	48	−1.5 ± 1.0 *	−0.7 ± 1.6 *	−0.9 (−1.6–−0.1)	0.032	0.65	−0.7 (−1.5–0.1)	0.078	0.27
3 month	48	−2.4 ± 1.5 *	−1.4 ± 1.9 *	−1.0 (−2.0–0.0)	0.051	0.58	−0.8 (−1.9–0.4)	0.185	0.20
6 month	45	−2.7 ± 2.1 *	−1.3 ± 2.3 *	−1.4 (−2.8–−0.1)	0.037	0.65	−1.9 (−4.0–0.2)	0.075	0.28
∆ Systolic blood pressure									
3 month	48	−10.2 ± 12.7 *	−6.7 ± 10.5 *	−3.5 (−10.3–3.2)	0.298	0.30	−1.0 (−7.7–5.7)	0.756	0.04
6 month	45	−8.3 ± 9.7 *	−5.3 ± 11.0 *	−3.0 (−9.3–3.2)	0.335	0.29	−6.1 (−15.2–3.0)	0.178	0.21
∆ Diastolic blood pressure									
3 month	48	−8.3 ± 6.8 *	−5.3 ± 8.2 *	−3.0 (−7.5–1.4)	0.173	0.40	−1.7 (−6.3–2.8)	0.437	0.11
6 month	45	−7.7 ± 8.8 *	−2.0 ± 7.0	−5.8 (−10.5–−1.0)	0.019	0.72	−6.4 (−13.2–0.5)	0.066	0.29
∆ HbA1c, %									
3 month	48	−0.4 ± 0.6 *	−0.3 ± 0.3 *	−0.1 (−0.4–0.1)	0.281	0.30	−0.1 (−0.2–0.0)	0.198	0.22
6 month	45	−0.3 ± 0.5 *	−0.1 ± 0.4	−0.1 (−0.4–0.2)	0.425	0.23	0.0 (−0.2–0.2)	0.764	0.00
∆ Fasting Blood Glucose, mmol/L									
3 month	48	−0.4 ± 0.7 *	−0.2 ± 0.5	−0.2 (−0.5–0.2)	0.347	0.27	−0.1 (−0.4–0.2)	0.389	0.11
6 month	45	−0.3 ± 0.7	−0.3 ± 0.7 *	−0.0 (−0.4–0.4)	0.970	0.01	0.2 (−0.2–0.6)	0.246	0.16
∆ Total cholesterol, mmol/L									
3 month	48	−0.45 ± 0.59 *	−0.21 ± 0.65	−0.23 (−0.60–0.13)	0.202	0.39	−0.32 (−0.75–0.10)	0.131	0.22
6 month	45	−0.54 ± 0.76 *	−0.05 ± 0.56	−0.48 (−0.88–−0.09)	0.019	0.73	−0.32 (−0.92–0.28)	0.283	0.17
∆ HDL cholesterol, mmol/L									
3 month	71	−0.06 ± 0.15	−0.08 ± 0.13 *	0.03 (−0.06–0.11)	0.533	0.14	0.03 (−0.06–0.11)	0.522	0.08
6 month	59	0.02 ± 0.16	0.03 ± 0.13	−0.01 (−0.09–0.08)	0.892	0.07	0.02 (−0.12–0.15)	0.814	0.04
∆ Triglycerides, mmol/L									
3 month	48	−0.19 ± 0.44	−0.23 ± 0.37 *	0.04 (−0.20–0.28)	0.730	0.10	0.07 (−0.18–0.33)	0.566	0.08
6 month	45	−0.26 ± 0.44 *	−0.13 ± 0.35	−0.12 (−0.36–0.11)	0.300	0.33	−0.25 (−0.56–0.07)	0.122	0.25
∆ LDL cholesterol, mmol/L									
3 month	48	−0.29 ± 0.49 *	−0.03 ± 0.61	−0.26 (−0.59–0.06)	0.110	0.47	−0.35 (−0.73–0.03)	0.066	0.27
6 month	45	−0.43 ± 0.67 *	−0.03 ± 0.52	−0.41 (−0.77–−0.05)	0.027	0.67	−0.23 (−0.75–0.30)	0.374	0.14
∆ Energy, kcal									
1 month	46	−282 ± 285 *	−679 ± 409 *	383 (176–590)	<0.001	1.13	280 (140–420)	**<0.001**	0.60
3 month	44	−441 ± 240 *	−604 ± 507 *	164 (−82–409)	0.185	0.41	33 (−147–213)	0.716	0.06
6 month	22	−460 ± 329 *	−593 ± 465 *	133 (−244–509)	0.471	0.33	20 (−318–358)	0.900	0.03
∆ Protein, g									
1 month	46	1.8 ± 15.1	−20.0 ± 24.5 *	20.0 (8.0–32.1)	0.002	1.07	18.9 (9.0–28.8)	**<0.001**	0.57
3 month	44	−12.3 ± 18.4 *	−14.3 ± 41.4	2.0 (−17.8–21.8)	0.842	0.06	−2.2 (−19.9–15.5)	0.802	0.04
6 month	22	−22.9 ± 19.3 *	−22.4 ± 28.9 *	−0.5 (−23.6–22.6)	0.964	0.02	2.5 (−17.2 to 22.1)	0.791	0.06
∆ Total fat, g									
1 month	46	−6.8 ± 16.3	−24.1 ± 23.0 *	16.5 (4.6–28.3)	0.007	0.86	13.1 (5.2–21.1)	**0.002**	0.49
3 month	44	−15.0 ± 12.9 *	−20.8 ± 30.6 *	5.8 (−8.7–20.3)	0.425	0.25	−0.2 (−11.3–11.0)	0.978	0.01
6 month	22	−11.8 ± 13.4 *	−21.2 ± 28.3 *	9.5 (−11.8–30.7)	0.365	0.43	5.8 (−10.0–21.5)	0.443	0.17
∆ Saturated fat, g									
1 month	46	−6.2 ± 8.8 *	−10.6 ± 11.1 *	4.2 (−1.8–10.1)	0.166	0.44	2.0 (−1.2–5.2)	0.217	0.19
3 month	44	−7.8 ± 7.7 *	−9.3 ± 13.2 *	1.5 (−5.1–8.2)	0.646	0.14	−0.3 (−4.8–4.1)	0.878	0.02
6 month	22	−6.7 ± 5.0 *	−8.8 ± 11.3 *	2.1 (−6.4–10.5)	0.615	0.24	1.2 (−5.3–7.7)	0.691	0.09
∆ MUFA, g									
1 month	46	−0.7 ± 5.8	−8.0 ± 9.0 *	7.1 (2.6–11.6)	0.003	0.97	5.0 (2.1–8.0)	**0.001**	0.51
3 month	44	−4.9 ± 6.4 *	−8.6 ± 12.8 *	3.6 (−2.6–9.9)	0.246	0.36	1.0 (−3.5–5.4)	0.660	0.07
6 month	22	−1.7 ± 6.9	−8.6 ± 13.7 *	6.9 (−3.5–17.3)	0.184	0.63	2.2 (−5.2–9.7)	0.529	0.14
∆ PUFA, g									
1 month	46	0.4 ± 4.0	−3.0 ± 4.8 *	3.1 (0.5–5.7)	0.019	0.75	2.8 (0.4–5.2)	**0.022**	0.35
3 month	44	−1.8 ± 5.0	−3.3 ± 4.9 *	1.6 (−1.5–4.6)	0.307	0.32	1.8 (−1.1–4.7)	0.208	0.19
6 month	22	−1.0 ± 4.7	−2.5 ± 5.3	1.5 (−3.1–6.1)	0.493	0.31	1.3 (−1.4–4.0)	0.312	0.23
∆ Dietary Cholesterol, mg									
1 month	46	−7.73 ± 185.69	−85.58 ± 200.99	69.59 (−45.79–184.96)	0.231	0.40	66.66 (−21.27–154.59)	0.133	0.23
3 month	44	−119.63 ± 190.38 *	−30.85 ± 269.06	−88.79 (−231.86–54.29)	0.217	0.38	−99.49 (−210.70–11.73)	0.078	0.27
6 month	22	−111.94 ± 218.84	−74.81 ± 185.63	−37.14 (−217.66–143.39)	0.672	0.18	31.01 (−87.47–149.50)	0.579	0.12
∆ Trans fat, g									
1 month	46	−0.1 ± 0.1 *	−0.2 ± 0.3 *	0.1 (−0.0–0.2)	0.088	0.53	0.1 (0.0–0.1)	0.038	0.51
3 month	44	−0.1 ± 0.3	−0.1 ± 0.4	0.1 (−0.1–0.3)	0.358	0.27	0.0 (−0.1–0.2)	0.735	0.00
6 month	22	−0.1 ± 0.3	−0.1 ± 0.2 *	0.0 (−0.2–0.2)	0.836	0.09	0.1 (0.0–0.1)	0.045	0.76
∆ Carbohydrate, g									
1 month	46	−56.6 ± 53.4 *	−95.5 ± 41.4 *	38.8 (10.6–67.1)	0.008	0.81	18.4 (2.4–34.3)	**0.025**	0.34
3 month	44	−66.6 ± 45.3 *	−92.4 ± 33.6 *	25.8 (1.7–49.9)	0.036	0.65	10.0 (−2.2–22.2)	0.106	0.25
6 month	22	−63.6 ± 60.0 *	−77.0 ± 45.2 *	13.5 (−33.3–60.2)	0.555	0.25	−9.0 (−48.1–30.0)	0.623	0.11
∆ Net Carbohydrate, g									
1 month	46	−61.6 ± 54.1 *	−93.5 ± 40.4 *	32.2 (3.9–60.5)	0.027	0.67	11.1 (−4.2–26.4)	0.152	0.22
3 month	44	−66.8 ± 47.0 *	−90.4 ± 30.1 *	23.7 (−0.1–47.5)	0.051	0.60	8.9 (−3.0–20.9)	0.138	0.23
6 month	22	−62.5 ± 60.7 *	−75.1 ± 41.3 *	12.6 (−32.6–57.8)	0.568	0.24	−6.4 (−43.0–30.2)	0.710	0.08
∆ Sugar, g									
1 month	46	−14.5 ± 19.6 *	−24.4 ± 16.6 *	9.8 (−1.0–20.4)	0.073	0.54	6.9 (1.9–12.0)	**0.009**	0.41
3 month	44	−17.7 ± 20.6 *	−23.1 ± 15.5 *	5.4 (−5.6–16.4)	0.328	0.30	1.8 (−3.5–7.1)	0.489	0.10
6 month	22	−17.9 ± 19.2 *	−17.1 ± 20.2 *	−0.8 (−18.7–17.1)	0.925	0.04	−0.7 (−12.9–11.6)	0.909	0.03
∆ Fiber, g									
1 month	46	5.0 ± 6.2 *	−2.0 ± 5.3	6.8 (3.5–10.1)	<0.001	1.22	6.7 (3.1–10.4)	**<0.001**	0.55
3 month	44	0.5 ± 6.9	−1.9 ± 6.6	2.4 (−1.8–6.5)	0.253	0.35	0.7 (−3.6–4.9)	0.757	0.05
6 month	22	−0.7 ± 2.6	−2.0 ± 8.9	1.3 (−5.1–7.7)	0.669	0.20	−1.8 (−10.0–6.4)	0.647	0.10
∆ Sodium, mg									
1 month	46	−83 ± 779	−789 ± 963 *	652 (143–1162)	0.013	0.81	671 (216–1126)	**0.005**	0.44
3 month	44	−471 ± 764 *	−707 ± 1419 *	237 (−467–940)	0.501	0.21	231 (−333–794)	0.410	0.13
6 month	22	−740 ± 780 *	−990 ± 1074	250 (−625–1125)	0.558	0.27	121 (−481–724)	0.668	0.09

Data expressed as mean ± SD. BMI, Body Mass Index; HbA1c, Glycated Hemoglobin; HDL, High-density Lipoprotein; LDL, Low-density Lipoprotein; MUFA, Monounsaturated fatty acid; PUFA, Polyunsaturated fatty acid. ^a^ adjusted for gender, age, change in step count and baseline value of the outcome. ^b^ adjusted for gender, age and change in step count. * Significant within group changes *p* values after Benjamini–Hochberg correction with false discovery rate at 0.20 and *n* = 122. Significant adjusted *p* values after Benjamini–Hochberg correction with false discovery rate at 0.20 and *n* = 64 in bold.

**Table 3:** Odds ratio of the HKD-RTE group achieving weight loss ≥5% at 1, 3, and 6 months in comparison to the HKD group.

	Weight Loss < 5%	Weight Loss ≥ 5%	OR (95% CI) ^a^	*p* Value
1 month				
HKD-RTE	13 (59.1%)	9 (40.9%)	4.3 (0.9–20.2)	0.069
HKD	21 (80.8%)	5 (19.2%)	1.0	
3 month				
HKD-RTE	8 (36.4%)	14 (63.6%)	1.02 (0.24–4.41)	0.975
HKD	11 (42.3%)	15 (57.7%)	1.0	
6 month				
HKD-RTE	8 (38.1%)	13 (61.9%)	1.4 (0.2–8.4)	0.714
HKD	11 (45.8%)	13 (54.2%)	1.0	

^a^ adjusted for gender, age and change in step count.

**Table 4:** Association between first month HKD-RTE meal compliance and weight loss (kg and %) at 1, 3, and 6 months in the HKD-RTE group.

	Unadjusted		Adjusted	
	Mean Difference (95% CI)	*p*-Value	Mean Difference (95% CI)	*p*-Value
1 month ∆ Weight, kg				
Percentage compliance	−0.05 (−0.10–0.01)	0.091	−0.05 (−0.12–0.02) ^a^	0.153 ^a^
3 month ∆ Weight, kg				
Percentage compliance	−0.083 (−0.163–0.002)	**0.044**	−0.109 (−0.218–0.001) ^a^	**0.049 ^a^**
6 month ∆ Weight, kg				
Percentage compliance	−0.14 (−0.25–−0.02)	**0.021**	−0.22 (−0.52–0.07) ^a^	0.110 ^a^
1 month ∆ Weight, %				
Percentage compliance	−0.06 (−0.12–0.00)	0.065	−0.04 (−0.12–0.04) ^b^	0.285 ^b^
3 month ∆ Weight, %				
Percentage compliance	−0.11 (−0.20–−0.01)	**0.030**	−0.10 (−0.23–0.02) ^b^	0.089 ^b^
6 month ∆ Weight, %				
Percentage compliance	−0.17 (−0.30–−0.04)	**0.016**	−0.22 (−0.49–0.05) ^b^	0.091 ^b^

^a^ adjusted for age, gender, change in step count and baseline weight. ^b^ adjusted for age, gender and change in step count. Bold highlights the statistically significant result.

## Data Availability

The data presented in this study are available on request from the corresponding author due to privacy reasons.
